# Autoimmunity plays a role in the onset of diabetes after 40 years of age

**DOI:** 10.1007/s00125-019-05016-3

**Published:** 2019-11-11

**Authors:** Olov Rolandsson, Christiane S. Hampe, Stephen J. Sharp, Eva Ardanaz, Heiner Boeing, Guy Fagherazzi, Francesca Romana Mancini, Peter M. Nilsson, Kim Overvad, Maria-Dolores Chirlaque, Miren Dorronsoro, Marc J. Gunter, Rudolf Kaaks, Timothy J. Key, Kay-Tee Khaw, Vittorio Krogh, Tilman Kühn, Domenico Palli, Salvatore Panico, Carlotta Sacerdote, Maria-José Sánchez, Gianluca Severi, Annemieke M. W. Spijkerman, Rosario Tumino, Yvonne T. van der Schouw, Elio Riboli, Nita G. Forouhi, Claudia Langenberg, Nicholas J. Wareham

**Affiliations:** 1 Department of Public Health and Clinical Medicine, Family Medicine, Umeå University, 901 87 Umeå, Sweden; 2 Department of Medicine, Division of Metabolism, Endocrinology and Nutrition, University of Washington, Seattle, WA USA; 3 MRC Epidemiology Unit, University of Cambridge School of Clinical Medicine, Institute of Metabolic Science, Cambridge, UK; 4 Navarre Public Health Institute, Pamplona, Spain; 5 Consortium for Biomedical Research in Epidemiology and Public Health (CIBER Epidemiología y Salud Publica), Madrid, Spain; 6 IdiSNA, Navarra Institute for Health Research, Pamplona, Spain; 7 Department of Epidemiology, German Institute of Human Nutrition Potsdam-Rehbruecke, Nuthetal, Germany; 8 CESP, Faculty of Medicine – University Paris-South, Faculty of Medicine Inserm U1018, University Paris-Saclay, Villejuif, France; 9 Department of Clinical Sciences, Clinical Research Center, Skåne University Hospital, Lund University, Malmö, Sweden; 10 Department of Public Health, Aarhus University, Aarhus, Denmark; 11 Department of Cardiology, Aalborg University Hospital, Aalborg, Denmark; 12 Department of Epidemiology, Murcia Regional Health Council, IMIB-Arrixaca, Murcia, Spain; 13 Public Health Division of Gipuzkoa, Basque Government, San Sebastian, Spain; 14 Instituto BIO-Donostia, Basque Government, San Sebastian, Spain; 15 International Agency for Research on Cancer, Lyon, France; 16 Division of Cancer Epidemiology, German Cancer Research Center (DKFZ), Heidelberg, Germany; 17 Cancer Epidemiology Unit, Nuffield Department of Population Health, University of Oxford, Oxford, UK; 18 Department of Public Health and Primary Care, University of Cambridge, Addenbrooke’s Hospital, Cambridge, UK; 19 Epidemiology and Prevention Unit, Fondazione IRCCS Istituto Nazionale dei Tumori, Milan, Italy; 20 Institute for Cancer Research, Prevention and Clinical Network – ISPRO, Florence, Italy; 21 Dipartimento di Medicina Clinica e Chirurgia, Federico II University, Naples, Italy; 22 Unit of Cancer Epidemiology, Azienda Ospedaliera Universitaria (AOU) Citta’ della Salute e della Scienza Hospital–University of Turin and Center for Cancer Prevention (CPO), Torino, Italy; 23 Andalusian School of Public Health, Granada, Spain; 24 Instituto de Investigación Biosanitaria de Granada (ibs.GRANADA), Universidad de Granada, Granada, Spain; 25 Inserm, Center for Research in Epidemiology and Population Health (CESP), Université Paris-Sud, Université Paris-Saclay, University of Versailles Saint-Quentin-en-Yvelines (UVSQ) Gustave Roussy, Villejuif, France; 26 Facultés de Medicine, Université Paris-Sud, Université Paris-Saclay, University of Versailles Saint-Quentin-en-Yvelines (UVSQ) Gustave Roussy, Villejuif, France; 27 National Institute for Public Health and the Environment (RIVM), Bilthoven, the Netherlands; 28 Cancer Registry and Histopathology Department, ‘Civic – M.P. Arezzo’ Hospital, Ragusa, Italy; 29 Associazone Iblea per la Ricerca Epidemiologica – Organizazione Non Lucrativa di Utilità Sociale, Ragusa, Italy; 30 Julius Center for Health Sciences and Primary Care, University Medical Center Utrecht, Utrecht, the Netherlands; 31 School of Public Health, Imperial College London, London, UK

**Keywords:** Autoantibody, Autoimmunity, Genetic risk score, Incident diabetes, Type 1 diabetes, Type 2 diabetes

## Abstract

**Aims/hypothesis:**

Type 1 and type 2 diabetes differ with respect to pathophysiological factors such as beta cell function, insulin resistance and phenotypic appearance, but there may be overlap between the two forms of diabetes. However, there are relatively few prospective studies that have characterised the relationship between autoimmunity and incident diabetes. We investigated associations of antibodies against the 65 kDa isoform of GAD (GAD65) with type 1 diabetes and type 2 diabetes genetic risk scores and incident diabetes in adults in European Prospective Investigation into Cancer and Nutrition (EPIC)-InterAct, a case-cohort study nested in the EPIC cohort.

**Methods:**

GAD65 antibodies were analysed in EPIC participants (over 40 years of age and free of known diabetes at baseline) by radioligand binding assay in a random subcohort (*n* = 15,802) and in incident diabetes cases (*n* = 11,981). Type 1 diabetes and type 2 diabetes genetic risk scores were calculated. Associations between GAD65 antibodies and incident diabetes were estimated using Prentice-weighted Cox regression.

**Results:**

GAD65 antibody positivity at baseline was associated with development of diabetes during a median follow-up time of 10.9 years (HR for GAD65 antibody positive vs negative 1.78; 95% CI 1.43, 2.20) after adjustment for sex, centre, physical activity, smoking status and education. The genetic risk score for type 1 diabetes but not type 2 diabetes was associated with GAD65 antibody positivity in both the subcohort (OR per SD genetic risk 1.24; 95% CI 1.03, 1.50) and incident cases (OR 1.97; 95% CI 1.72, 2.26) after adjusting for age and sex. The risk of incident diabetes in those in the top tertile of the type 1 diabetes genetic risk score who were also GAD65 antibody positive was 3.23 (95% CI 2.10, 4.97) compared with all other individuals, suggesting that 1.8% of incident diabetes in adults was attributable to this combination of risk factors.

**Conclusions/interpretation:**

Our study indicates that incident diabetes in adults has an element of autoimmune aetiology. Thus, there might be a reason to re-evaluate the present subclassification of diabetes in adulthood.

**Electronic supplementary material:**

The online version of this article (10.1007/s00125-019-05016-3) contains peer-reviwed but unedited supplementary material, which is available to authorised users.

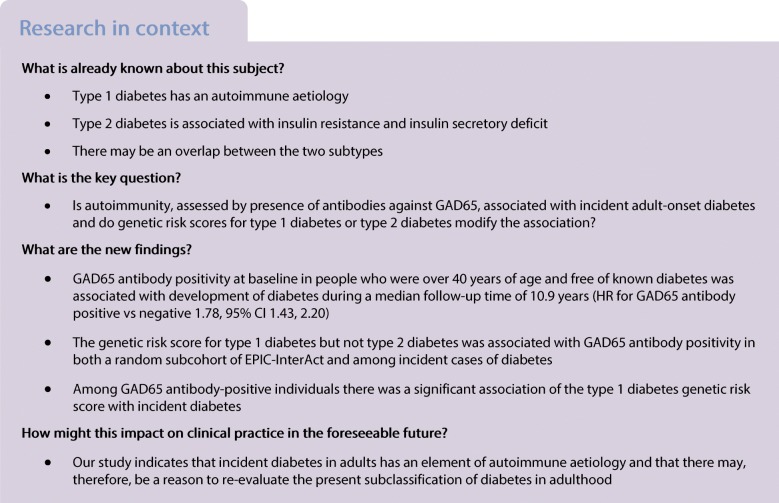



## Introduction

Diabetes mellitus has classically been thought to have two major aetiological subtypes: type 1 diabetes, which is characterised by autoimmune destruction of beta cells with subsequent insulin deficiency, and type 2 diabetes, which is associated with insulin resistance and a relative insulin secretory deficit [1]. In type 1 diabetes the autoimmune response manifests itself in T cell reactivity and autoantibody responses directed against at least four beta cell autoantigens, including the 65 kDa isoform of GAD (GAD65) [2]. Although the pathogenesis of type 2 diabetes is thought to be different from that of type 1 diabetes, there may be some overlap; however, the evidence for this is limited [3]. Associations of beta cell autoimmunity, as assessed by presence of GAD65 antibodies, with insulin requirement and diabetes complications have been investigated in prevalent adult-onset diabetes [4–14]. In contrast, there are only a few population-based prospective studies exploring the association between autoimmunity and incident diabetes. Moreover, while some of these studies reported an association [15, 16], others have been inconclusive [17–19]. In a recent meta-analysis Koopman et al reported that the pooled risk estimate of incident type 2 diabetes for GAD65 antibody positivity, compared with GAD65 antibody negativity, was 3.36 (95% CI 1.9, 5.9) [20]. However, there was significant heterogeneity between the studies in the meta-analysis.

Susceptibility for development of GAD65 antibody and type 1 diabetes is at least in part explained by risk alleles located within the HLA region on chromosome 6 [21–23]. We have previously shown that specific HLA haplotypes are associated with GAD65 antibody positivity [23]. It is unknown whether a possible association between GAD65 antibodies and adult-onset diabetes is explained by these HLA haplotypes. It has also been suggested that type 2 diabetes risk alleles are associated with a subset of adult-onset diabetes characterised by presence of GAD65 antibodies at or around the time of diagnosis [24–26], but this association may be affected by the level of the cut-off used to define presence of autoantibodies [27].

Thus, we aimed to investigate the association between GAD65 antibody levels measured using a competition assay, which increases the precision of the assessment of autoantibody positivity, and incident adult-onset diabetes in a large, multi-centre, population-based prospective study in eight European countries in people who were over 40 years of age and free of known diabetes at baseline. In addition, we investigated whether genetic risk scores (GRSs) for type 1 diabetes and type 2 diabetes are associated with GAD65 antibody positivity, and the potential for either of these risk scores to modify the association of GAD65 antibodies with incident adult-onset diabetes.

## Methods

### Population

The design and methods of the European Prospective Investigation into Cancer and Nutrition (EPIC)-InterAct case-cohort study have previously been described [28]. A total of 340,234 EPIC participants, who were over the age of 40 and free of known diabetes at baseline, in eight of the ten EPIC study countries (26 centres) were followed up for 3.99 million person-years (median follow-up 10.9 years), during which 12,403 incident cases of diabetes were ascertained and verified. The mean (SD) age at diagnosis was 62.3 (7.8) years in men and 62.6 (8.2) years in women [28]. Ascertainment of incident diabetes involved multiple sources of evidence including self-report, linkage to primary care registers, secondary care registers, medication use (drug registers), hospital admissions and mortality data, with a minimum of two data sources being required to confirm the diagnosis. Cases in Denmark and Sweden were not ascertained by self-report but identified via local and national diabetes and pharmaceutical registers, and hence all ascertained cases were considered to be verified. Information from any follow-up visit or external evidence with a date later than the baseline visit was used. Follow-up was censored at the date of diagnosis, 31 December 2007 or the date of death, whichever occurred first. A centre-stratified subcohort of 16,835 (4.9% of the entire EPIC cohort) individuals was selected at random [28]. We excluded 548 individuals with known prevalent diabetes and 133 with unknown diabetes status at baseline. We also excluded 422 cases and 352 subcohort participants (of whom 34 were incident cases) with insufficient sample volume for GAD65 antibody measurement, resulting in 15,802 subcohort participants and 11,981 incident cases being included in the analysis.

All study participants gave informed consent, and the investigation has been carried out in accordance with the Declaration of Helsinki as revised in 2008.

### GAD65 antibody measurement

Blood samples were drawn at the time of participation in EPIC, at which time all participants were free of known diabetes. Blood plasma was prepared and stored at −196°C in liquid nitrogen at the coordinating centre at the International Agency for Research into Cancer (IARC) in Lyon, France, or in liquid nitrogen in local biorepositories except for Umeå, where −80°C freezers were used [28].

The samples had been subject to at least two freeze-thaw cycles before being analysed for GAD65 antibody. Recombinant ^[35]^S-GAD65 was produced in an in vitro coupled transcription and translation system with SP6 RNA polymerase and nuclease-treated rabbit reticulocyte lysate (Promega, Madison, WI, USA) as previously described [29]. The WHO standard [30] was included and used to express immunoglobulin binding levels in relative units.

**Fig. 1:**
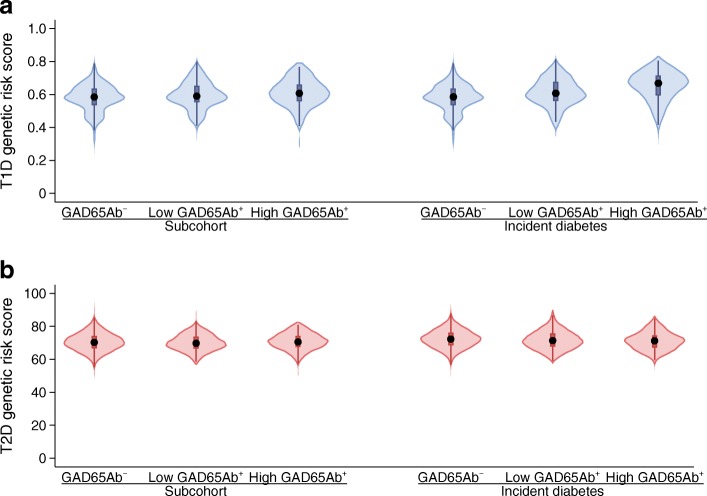
Violin plots of risk scores according to GAD65 antibody status for (**a**) type 1 diabetes and (**b**) type 2 diabetes in the EPIC-InterAct subcohort and incident diabetes cases. *n* values in (**a**) are: 12,526 (subcohort, GAD65Ab^−^), 149 (subcohort, low GAD65Ab^+^), 104 (subcohort, high GAD65Ab^+^), 9442 (incident diabetes, GAD65Ab^−^), 139 (incident diabetes, low GAD65Ab^+^), and 196 (incident diabetes, high GAD65Ab^+^). *n* values in (**b**) are: 12,249 (subcohort, GAD65Ab^−^), 146 (subcohort, low GAD65Ab^+^), 99 (subcohort, high GAD65Ab^+^), 9126 (incident diabetes, GAD65Ab^−^) and 135 (incident diabetes, low GAD65Ab^+^), 192 (incident diabetes, high GAD65Ab^+^). ‘High’ GAD65Ab^+^ is defined as GAD65 antibody ≥167.5 U/ml and ‘low’ GAD65Ab^+^ is defined as GAD65 antibody <167.5 U/ml. GAD65Ab^+^, GAD65 antibody positive; GAD65Ab^−^, GAD65 antibody negative; T1D, type 1 diabetes; T2D, type 2 diabetes

To determine the cut-off for GAD65 antibody positivity, we used a competition assay employing recombinant human GAD65 (rhGAD65) (Diamyd Medical, Stockholm, Sweden) as previously described [31]. A total of 900 serum samples were randomly selected across countries in the EPIC-InterAct study population. The samples were incubated with radiolabelled GAD65 in the absence or presence of rhGAD65 (200 ng/ml) or BSA (200 ng/ml). Samples in which binding to radiolabelled GAD65 was reduced by 50% in the presence of rhGAD65, but not BSA, were considered to be positive for GAD65-specific antibodies. We used 50% as a cut-off for successful competition as an approximation of the IC_50_. Given the low sample volume, we chose to use the competitor at the optimal concentration (200 ng/ml) found in previous experiments, in which we titrated the amount of rhGAD65 necessary to give maximal competition. In a variation of the traditional receiver operating characteristic (ROC) analysis, we plotted GAD65 antibody levels of samples that were competed by ≥50% against GAD65 antibody levels of samples that were competed by <50%. The area under the ROC curve was 0.97, indicating excellent predictive ability of the GAD65 antibody measurement. At a cut-off level of ≥65 U/ml, the measurement had 99% specificity and 85% sensitivity (electronic supplementary material [ESM] Fig. 1). Thereafter, all samples in the subcohort (*n* = 15,802) and incident cases (*n* = 11,981) were analysed for GAD65 antibodies in a radiobinding assay (RBA) as previously described [29].

### Measurement of covariates

Weight, height, and waist and hip circumferences were measured with participants not wearing shoes and in light clothing or underwear, as described previously [28]. BMI was calculated as weight/height squared (kg/m^2^). Waist circumference was measured either at the narrowest circumference of the torso or at the midpoint between the lower ribs and the iliac crest. Hip circumference was measured horizontally at the level of the largest lateral extension of the hips or over the buttocks. Anthropometric data were mostly self-reported in the Oxford centre, and waist and hip circumferences were not measured in the Umeå centre (*n* = 1845).

Standardised information on highest educational level (none, primary, technical, secondary or further education) and smoking status (current smoker, never a smoker or former smoker) was collected by questionnaire at baseline [28]. Physical activity was assessed using a brief questionnaire covering occupation and recreational activity, from which a validated physical activity index (inactive, moderately inactive, moderately active or active) was derived [32].

### Genetic analysis and GRS

Samples were processed for array-based genotyping if they had sufficient DNA that could be successfully genotyped on TaqMan (Thermo Fisher Scientific, Waltham, MA, USA) or Sequenom (San Diego, CA, USA) platforms and had sex chromosome genotypes concordant with self-reported sex. Samples that failed one genotyping round for reasons that did not relate to sample quality (e.g. signal intensity outliers or plates/arrays with an unusually high failure rate) were repeated. Samples were genotyped on the Illumina 660 W-Quad BeadChip, the Illumina HumanCoreExome-12 or the Illumina HumanCoreExome-24 (San Diego, CA, USA). Samples genotyped on the Illumina 660 W-Quad BeadChip were randomly selected from the available samples with the number of individuals selected per centre being proportional to the percentage of total cases in that centre. The Danish samples were not available for genotyping at this stage. Genotyping was carried out at the Wellcome Trust Sanger Institute. Most of the remaining non-Danish samples were genotyped on the Illumina HumanCoreExome-12 at Cambridge Genomic Services in the University of Cambridge Department of Pathology. Finally, the Danish samples and repeat samples due to poor genotyping were genotyped on the Illumina HumanCoreExome-24 also at Cambridge Genomic Services. Sample quality control criteria varied slightly by array but included call rate (<95.4% in Illumina 660, <98% in core exome arrays), X chromosome heterozygosity concordance with self-reported sex, outliers for heterozygosity and concordance with previous genotyping results.

From the genome-wide array data, we calculated a type 1 diabetes GRS as a weighted average of 33 SNPs, including five HLA variants. The relevant SNPs and their individual associations with GAD65 antibody positivity are described in ESM Table 1 [33]. We also calculated a type 2 diabetes GRS as a weighted average of 68 SNPs from a DIAbetes Genetics Replication And Meta-analysis (DIAGRAM) consortium publication [34].

### Statistical analyses

Baseline characteristics of the analysis sample were summarised by GAD65 antibody status (negative/positive), separately within the subcohort and incident diabetes cases, using means and standard deviations for continuous variables (except for GAD65 antibody level, which had a skewed distribution so the median and interquartile range were used) and percentages for categorical variables.

The association between GAD65 antibody status (positive/negative) and incident diabetes was estimated using Prentice-weighted Cox regression, which is appropriate for estimating association in a case-cohort study. We fitted models within each country and the estimated HRs were combined across countries using random effects meta-analysis. We fitted three models including the following covariates: Model 1—age (as underlying time scale), sex and centre; Model 2—also including physical activity, smoking status and education; Model 3—also including family history of diabetes. In order to study the effects of high vs low GAD65 antibody levels, GAD65 antibody-positive individuals were further subdivided into those with GAD65 antibody equal to or above, and those with GAD65 antibody below, 167.5 U/ml, which is the median antibody level in the GAD65 antibody-positive group in this study. Prentice-weighted Cox regression was also used to test possible multiplicative interactions of GAD65 antibody status with: (1) sex; (2) BMI category; (3) waist/hip ratio (WHR) category (sex-specific tertiles); and (4) type 1 diabetes GRS tertile. The interactions with anthropometry measures were tested because of prior studies suggesting that adiposity could moderate the association of autoimmunity with diabetes [9, 10]. In this instance we fitted models to the overall dataset with adjustment for country, due to insufficient data within each country to obtain country-specific estimates. HRs and 95% CIs within each subgroup were calculated.

The associations of the type 1 diabetes GRS and the type 2 diabetes GRS with GAD65 antibody status (positive/negative) were estimated separately in the subcohort and the incident diabetes cases, using logistic regression adjusted for age and sex, since the prevalence of autoimmunity is associated with age and sex. Models were fitted within each country and estimated odds ratios combined across countries using random effects meta-analysis. ORs (and 95% CIs) per risk allele of each of the individual SNPs contributing to the type 1 diabetes GRS were also calculated in the subcohort using the same method.

The association between the type 1 diabetes GRS and incident diabetes, by GAD65 antibody status, was estimated using Prentice-weighted Cox regression as described above.

## Results

Three hundred and sixteen (2.0%) individuals in the subcohort and 413 (3.4%) incident cases were GAD65 antibody positive as defined by having a level of ≥65 U/ml (Table 1). Individuals who were GAD65 antibody positive in both the subcohort and incident cases tended to be leaner, and fewer of these individuals reported a family history of diabetes. There were no other major differences in baseline characteristics by GAD65 antibody status (Table 1). The distributions of the type 1 diabetes GRS and the type 2 diabetes GRS were similar when comparing individuals who were GAD65 antibody positive and negative, in both the subcohort and incident diabetes cases (Table 1, Fig. 1a,b).

**Table 1:** Baseline characteristics of the subcohort and incident diabetes cases; the EPIC-InterAct study (*N* = 27,039)

Characteristic	Subcohort		Incident diabetes cases	
	GAD65 antibody^−^*n* = 15,486	GAD65 antibody^+^*n* = 316	GAD65 antibody^−^*n* = 11,568	GAD65 antibody^+^*n* = 413
Women (%)	62.4	64.6	50.2	58.1
Age (years)	52.3 (9.1)	52.7 (9.3)	55.5 (7.6)	55.0 (8.4)
BMI (kg/m^2^)	26.0 (4.2)	25.8 (4.2)	29.8 (4.7)	28.4 (5.1)
WHR	0.85 (0.09)	0.85 (0.09)	0.92 (0.09)	0.89 (0.09)
Physical activity				
Inactive	23.7	25.2	30.1	33.3
Moderately inactive	33.5	35.8	32.9	28.0
Moderately active	22.7	19.5	20.3	20.2
Active	20.1	19.5	16.7	18.5
Highest schooling level				
None	7.7	10.3	10.1	8.6
Primary	33.0	34.3	41.9	40.9
Technical	23.3	20.8	23.6	21.9
Secondary	15.4	14.7	11.1	14.5
Further education	20.7	19.9	13.3	14.0
Smoking status				
Never	47.0	50.5	41.0	43.2
Former	27.1	25.2	31.2	28.0
Current	25.9	24.3	27.8	28.8
Family history of diabetes (yes)	18.7	14.5	36.3	29.5
GAD65 antibody (U/ml)	0.0 (0, 0)	133.3 (86.7, 336.1)	0.0 (0,0)	207.1 (99.3, 1000)
Type 1 diabetes risk score	0.6 (0.1)	0.6 (0.1)	0.6 (0.1)	0.6 (0.1)
Type 2 diabetes risk score	70.6 (5.7)	70.4 (5.2)	72.4 (5.7)	71.6 (5.7)

Values are presented as percentages for categorical variables and mean (SD) for continuous variables, except for GAD65 antibody, which has a skewed distribution and so median (interquartile range) is presented

**Fig. 2:**
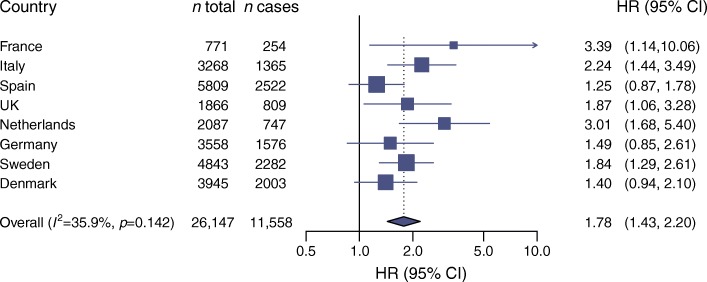
HRs for incident diabetes comparing GAD65 antibody-positive with GAD65 antibody-negative groups, by country, in the EPIC-InterAct study (Model 2, *n* = 26,147)

GAD65 antibody positivity was associated with a higher incidence of diabetes (Table 2) consistently across countries (Fig. 2). Of the potential confounders considered, only family history of diabetes had an impact on the HR, which increased from 1.78 (95% CI 1.43, 2.20) in Model 2 to 2.19 (95% CI 1.56, 3.07) in Model 3, which includes family history of diabetes. Data on family history of diabetes were not collected at some centres; fitting all three models to the sample with complete data on all covariates gave results similar to those above (ESM Table 2), suggesting the presence of negative confounding by family history. The association was markedly stronger in those with high GAD65 antibody levels than in those with lower levels, relative to those who were GAD65 antibody negative (Table 2), suggesting a potential dose–response effect. We undertook a sensitivity analysis to examine the effect of lowering the threshold for defining GAD65 antibody positivity from ≥65 U/ml to ≥40 U/ml. This would lead to an additional 255 incident diabetes cases being labelled as GAD65 antibody positive, but led to a weaker overall association between GAD65 antibody positivity and incident diabetes. A formal comparison of the effect size for the association with incident diabetes for the two definitions of GAD65 antibody positivity was not significant (*p* = 0.099).

**Table 2:** HRs for incident diabetes comparing GAD65 antibody-positive with GAD65 antibody-negative groups; the EPIC-InterAct study

Variable	Model 1					Model 2					Model 3				
	*N*	HR	Lower	Upper	*I*^2^ (%)	*N*	HR	Lower	Upper	*I*^2^ (%)	*N*	HR	Lower	Upper	*I*^2^ (%)
GAD65 antibody^+^ vs GAD65 antibody^−^	705 (+) 26,334 (−)	1.80	1.48	2.20	34	691 (+) 25,456 (−)	1.78	1.43	2.20	36	351 (+) 12,484 (−)	2.19	1.56	3.07	43
‘High’ GAD65 antibody^+^ vs GAD65 antibody^−^	353 (high)	2.46	1.91	3.17	18	341 (high)	2.43	1.85	3.18	19	211 (high)	2.73	1.58	4.70	62
‘Low’ GAD65 antibody^+^ vs GAD65 antibody^−^	352 (low)	1.32	1.01	1.72	25	350 (low)	1.28	0.99	1.66	18	140 (low)	1.74	1.21	2.50	3

Model 1: age (as underlying time scale), sex and centre; Model 2: age (as underlying time scale), sex, centre, physical activity, smoking status and education; Model 3: age (as underlying time scale), sex, centre, physical activity, smoking status, education and family history of diabetes

*I*^2^ represents percentage of variability due to heterogeneity between countries. ‘High’ GAD65 antibody^+^ is defined as GAD65 antibody ≥167.5 U/ml and ‘low’ GAD65 antibody^+^ is defined as GAD65 antibody <167.5 U/ml

Type 1 diabetes GRS, but not type 2 diabetes GRS, was associated with GAD65 antibody positivity in both the subcohort and incident diabetes cases (Table 3). Of the five SNPs in the type 1 diabetes GRS that are linked to HLA haplotypes, three were significantly associated with GAD65 antibody positivity in the subcohort after adjusting for age and sex (ESM Table 2). However, HLA haplotypes did not explain the association between GAD65 antibodies and incident diabetes. In a sensitivity analysis restricted to those individuals with complete relevant data (*n* = 10,893), the HR for incident diabetes in those positive for GAD65 antibodies compared with those individuals who were negative was 2.22 (95% CI 1.49, 3.31) without adjustment and 2.13 (95% CI 1.46, 3.11) with adjustment for a five HLA haplotype risk score. There was no association between any individual type 2 diabetes-associated SNP and GAD65 antibody level (ESM Table 3).

**Table 3:** Associations of type 1 diabetes and type 2 diabetes GRSs with GAD65 antibody positivity in the subcohort and incident diabetes cases; the EPIC-InterAct study

Variable	Subcohort					Incident diabetes cases				
	N	OR	Lower	Upper	I^2^ (%)	N	OR	Lower	Upper	I^2^ (%)
Type 1 diabetes GRS (per 1 SD)	12,779	1.24	1.03	1.50	46	9777	1.97	1.72	2.26	12
Type 2 diabetes GRS (per 1 SD)	11,054	0.97	0.85	1.12	0	8228	0.85	0.76	0.96	0

Associations are estimated from logistic regression, adjusted for age and sex. Models fit within each country; estimates combined across countries using random effects meta-analysis

There was no overall association between type 1 diabetes GRS and incident diabetes (HR 1.02 per SD type 1 diabetes GRS; 95% CI 0.99, 1.06) when adjusted for age, sex, physical activity, smoking status, education and BMI. However, there were significant interactions of GAD65 antibody status with BMI category (*p* = 0.001), WHR category (*p* <0.001) and type 1 diabetes GRS tertile (*p* <0.001), but no interaction of GAD65 antibody status with sex, on the hazard of diabetes. The associations between GAD65 antibody positivity and incident diabetes were strongest in the lowest categories of BMI and WHR, and in the highest tertile of type 1 diabetes GRS (Table 4).

**Table 4:** HRs (95% CIs) for incident diabetes comparing GAD65 antibody-positive with GAD65 antibody-negative groups within categories of BMI, WHR (sex-specific tertiles) and tertiles of type 1 diabetes GRS; the EPIC-InterAct study

Variable	GAD65 antibody^+^ vs GAD65 antibody^−^		
	*n*	HR	95% CI
BMI (kg/m^2^)			
<25	8251	3.35	(2.51, 4.47)
25 to <30	10,742	1.48	(1.14, 1.93)
≥30	6983	1.46	(1.02, 2.08)
WHR			
<0.91 (men), <0.77 (women)	5170	2.91	(2.00, 4.24)
0.91 to <0. 96 (men), 0.77 to <0.82 (women)	6770	2.37	(1.69, 3.30)
≥0.96 (men), ≥0.82 (women)	12,364	1.33	(1.05, 1.69)
Type 1 diabetes GRS			
<0.56	6939	1.25	(0.85, 1.84)
0.56 to <0.62	7066	1.14	(0.81, 1.60)
≥0.62	7258	2.52	(1.94, 3.29)

Models adjusted for age (as underlying timescale), sex, country, physical activity, smoking status and education. Tertiles calculated using distributions in the subcohort

Among GAD65 antibody-positive individuals there was a significant association of the type 1 diabetes GRS with incident diabetes (HR 2.42 per SD type 1 diabetes GRS; 95% CI 1.83, 3.21). There was no evidence of an association among GAD65 antibody-negative individuals (HR 1.00 per SD type 1 diabetes GRS; 95% CI 0.97, 1.04). In order to contextualise these interactions, we also analysed the interaction in terms of tertiles of the type 1 diabetes GRS rather than standard deviations. Among the 26,693 individuals with relevant data, 552 (2.1%) were GAD65 antibody positive and 284 (1.1%) were GAD65 antibody positive and also in the top tertile of the type 1 diabetes GRS. Of these 284 individuals, 196 developed incident diabetes and 88 were non-cases. The HR for incident diabetes in the GAD65 antibody-positive individuals comparing the top tertile of the type 1 diabetes GRS with the combination of the other two tertiles was 4.83 (95% CI 2.47, 9.43). When the exposed group was defined as those who were GAD65 antibody positive and in the top tertile of the type 1 diabetes GRS, the HR for incident diabetes compared with all other individuals was 3.23 (95% CI 2.10, 4.97). From this it follows that the population attributable fraction, the theoretical fraction of all cases attributable to having GAD65 antibodies and high type 1 diabetes genetic risk, was 1.8%. Baseline characteristics of the subcohort stratified by both GAD65 antibody status and tertile of the type 1 diabetes GRS (high vs middle/low) are summarised in ESM Table 4. In a further post hoc analysis, using ≥40 U/ml rather than ≥65 U/ml as the threshold for antibody positivity, the HR for incident diabetes comparing those who were antibody positive and in the top tertile of the type 1 diabetes GRS was 2.05 (95% CI 1.60, 2.62); the population attributable fraction was 1.7%.

## Discussion

This large, prospective, population-based European study found a significant association between GAD65 antibody positivity and development of incident diabetes after the age of 40 years in individuals free of known diabetes at baseline in eight European countries. A GRS for type 1 diabetes, but not type 2 diabetes, was associated with GAD65 antibody positivity. There was no overall association of the type 1 diabetes GRS with incident diabetes, but, in the subgroup of individuals who were positive for GAD65 antibodies, the type 1 diabetes GRS was strongly associated with incident diabetes. From these data we estimate that just under 2% of all cases of adult-onset diabetes are attributable to the combination of having a high genetic risk for type 1 diabetes and being positive for GAD65 antibodies.

Our study provides definitive evidence of the association between GAD65 antibodies and incident diabetes in European populations, by virtue of the size of the population and extensive follow-up (nearly 4 million person-years), as well as the use of a specific method for defining antibody positivity. An association between higher GAD65 antibody levels and incident diabetes in adults has been suggested previously [15, 16]. However, the Botnia study was not population-based and was based on relatives of individuals with type 2 diabetes [15], and our previous investigation of adult participants in the Västerbotten Intervention Programme was extremely small and produced estimates of the effect size with wide confidence intervals [16]. A null association between GAD65 antibodies and development of type 2 diabetes reported in Northern Italy may reflect the small size of this study, which was underpowered to detect a true association [18]. The Diabetes Prevention Program also reported no association between GAD65 antibodies and development of type 2 diabetes [19]. However, the follow-up of the Diabetes Prevention Program cohort was very short (3.2 years) and included individuals of various ethnicities with different risks of diabetes, both of which may explain the null overall association. Finally, Sorgjerd et al reported that none of the 349 individuals that developed type 2 diabetes in The Nord-Trøndelag Health Study (HUNT) 3 were positive for GAD65 antibodies in HUNT2 [12]. However, these individuals represented only 23% of all individuals that developed type 2 diabetes in HUNT3, and type 2 diabetes was classified as absence of GAD65 antibodies. Notably, we did not analyse the GAD65 antibody status at time of diagnosis and assume that a significant portion of these individuals will present GAD65 antibodies at diagnosis. Finally, development of type 2 diabetes in HUNT3 was analysed in a retrospective manner, while our study was conducted prospectively. Therefore, outcomes of these studies are difficult to compare. Our study was restricted to European populations, so we cannot shed any light on ethnic differences in the links between these antibodies and incident diabetes.

Our finding that type 1 diabetes GRS was associated with GAD65 antibody positivity in the subcohort is in line with the study by Mishra et al, which was based on prevalent latent autoimmune diabetes in adults (LADA) cases and non-diabetic control individuals [27]. The HLA types included in our analysis were selected on the basis of their known association with type 1 diabetes as part of an overall type 1 diabetes GRS [35]. The association was mainly explained by the SNPs for HLA-DR3/DR, HLA-DR3/DR4 and HLA-DQB1*0302 (also named HLA-DQ8). We reported in a preliminary smaller study [23] that the presence of HLA-DR3 was associated with GAD65 antibody levels in a non-diabetic population. The same pattern of association for HLA-DR3 was also observed in a study of Finnish school children, where, in addition, HLA-DQB1*0302 was also associated with GAD65 antibody positivity [21]. However, both of these small studies were cross-sectional and were unable to examine whether the individuals with antibodies and type 1 diabetes risk alleles were more or less likely to develop diabetes later in life.

This study indicates that autoimmunity plays a role in the aetiology of adult-onset diabetes, irrespective of how the people were classified clinically. We estimate that 1.8% of incident diabetes cases in adulthood are attributable to the combination of having a high genetic risk for type 1 diabetes and having GAD65 antibodies. Our interpretation is that there is an overlap in the aetiology in adult diabetes. It has been shown that presence of GAD65 antibodies predicts future beta cell dysfunction which could be the result of an autoimmune attack on the beta cells (reviewed in [3]), even in people classified as having type 2 diabetes. In some cases, the autoimmune attack does not lead to a total beta cell failure. The result of the autoimmune attack is very dependent on the titres of autoantibodies, presence of multiple antibodies and genetic risk [3]. This notion of a scale of the effect of the autoimmune attack was further strengthened by the finding that transient GAD65 antibodies [12] led to earlier onset of diabetes and contributed to a relative beta cell dysfunction. In line with our hypothesis that there is an overlap between aetiologies in adult-onset diabetes, autoimmunity as defined by insulitis assessed by histological investigation was found in pancreases of people diagnosed with type 2 diabetes [36]. However, we can only speculate about the mechanism behind the association, although it has been reported that insulin resistance could be present also among those with GAD65 antibodies [11]. The mechanism could be that insulin resistance increases the secretion of insulin from the beta cells, thereby exposing more antigen (i.e. GAD65, which is co-excreted with insulin from the vesicles in the beta cells) to the immune system. In people with a predisposition for autoimmunity, this autoimmune response towards the beta cells might lead to beta cell dysfunction and/or beta cell death. This hypothesis is supported by findings in an experimental study design [37] that reported an association between GAD65 antibodies and a decrease in maximal insulin secretory capacity in people without diabetes. This suggests that the presence of GAD65 antibodies is a pancreatic marker of a subclinical autoimmune process that could lead to insulin deficiency and subsequently type 2 diabetes.

Taken together, there might be a reason to re-evaluate the present subclassification of diabetes in adulthood [1]. This re-evaluation might also lead to studies aimed at the prevention of diabetes, while taking the presence of autoimmunity into account, and to studies trying to optimise the treatment of adult-onset diabetes.

Our study has limitations. The presence of GAD65 antibody at baseline suggests an autoimmune component in the aetiology of adult-onset diabetes over the age of 40. We deliberately sought to use this descriptive term to avoid forcing individuals into predetermined diagnostic categories, since this has the potential to create circular arguments [38, 39]. We did not have access to GAD65 antibody measurements at the time of diabetes diagnosis but would anticipate that antibodies measured before the diagnosis of diabetes would persist, as previous studies have demonstrated [40, 41]. Several features of our study add to the strength of this analysis. First, all samples were analysed in the same laboratory. The samples were blinded to case or non-case status and samples of participants from different countries were analysed together to minimise assay variation. Second, the cut-off was determined in a subsample of 900 samples, based on specificity of binding, rather than setting an arbitrary cut-off level based on percentiles. Third, the same cut-off level was applied to all samples.

In conclusion, in this large, population-based prospective study, we found that GAD65 antibody positivity was associated with the incidence of diabetes after the age of 40 years. A GRS for type 1 diabetes but not type 2 diabetes was associated with GAD65 antibody positivity and with the incidence of diabetes in those who were GAD65 antibody positive. Our data suggest that incident diabetes in adults includes an element of autoimmune aetiology, which warrants further prospective studies into the risk factors associated. Moreover, future studies with a different design should investigate the implications of this finding for subclassification of incident diabetes in adults, selection of primary treatment modalities and frequency of risk factor assessment to enhance prognosis.

## Electronic supplementary material

ESM (PDF 230 kb)

## Data Availability

The datasets generated during and/or analysed during the current study are available from the corresponding author on reasonable request.

## Abbreviations

EPIC: European Prospective Investigation into Cancer and Nutrition
GAD65: 65 kDa isoform of GAD
GRS: Genetic risk score
HUNT: The Nord-Trøndelag Health Study
rhGAD65: Recombinant human GAD65
ROC: Receiver operating characteristic
