# Supplementary material for: Autoimmunity plays a role in the onset of diabetes after 40 years of age
Source: Diabetologia. 2019 Nov 11;63(2):266–77. doi: 10.1007/s00125-019-05016-3 (PMC6946728; doi:10.1007/s00125-019-05016-3)

ESM Table 1. Associations of type 1 diabetes SNPs/variants with GAD65 antibody positivity in the EPIC-InterAct subcohort.

SNPs/variants	N	OR	Lower 95% CI	Upper 95% CI
rs11755527	12779	1.12	0.94	1.34
rs1264813	12692	0.89	0.66	1.19
rs1465788	12625	1.01	0.83	1.24
rs17574546	12779	1.05	0.84	1.30
rs1893217	12693	1.10	0.87	1.39
rs1990760	12779	1.07	0.89	1.28
rs2281808	12779	0.90	0.75	1.08
rs3129889	12715	1.24	0.86	1.78
rs3788013	12779	1,07	0.90	1.28
rs3825932	12779	1.17	0.96	1.42
rs4948088	12755	0.86	0.56	1.31
rs5753037	12779	1.02	0.85	1.23
rs689	12779	1.10	0.90	1.34
rs7202877	12707	0.87	0.61	1.24
rs9388489	12779	1.17	0.98	1.40
rs10509540	12779	0.93	0.76	1.13
rs11594656	12610	1.06	0.86	1.30
rs12708716	12584	1.05	0.81	1.38
rs12722495	12779	1.01	0.68	1.48
rs2069762	12779	1.18	0.96	1.45
rs2292239	12580	1.12	0.93	1.34
rs2395029	12681	0.74	0.44	1.25
**rs2476601**	**12779**	**1.47**	**1.12**	**1.92**
rs3024505	12779	1.01	0.79	1.29
rs3087243	12779	0.89	0.72	1.11
rs425105	12682	0.88	0.69	1.11

rs4788084	12779	1.08	0.90	1.29
rs763361	12779	1.05	0.81	1.35
**DR3/DR3**	**12720**	**4.07**	**2.01**	**8.26**
**DR3/DR4-DQ8**	**12742**	**2.42**	**1.32**	**4.43**
DR3/X	12733	1.18	0.86	1.63
**DR4-DQ8/DR4-DQ8**	**12755**	**3.93**	**1.78**	**8.68**
DR4-DQ8/X	12732	0.83	0.47	1.43

Odds ratios (lower and upper 95% CI) are per 1 unit (i.e. risk allele) of each SNP/variant.
Associations are estimated from logistic regression, adjusted for age and sex. Results highlighted in bold font are statistically significant at the 5% level. DR3 refers to HLA-DRB1*03 and DR4-DQ8 to HLA-DRB1*04 with DQB1*03:02.

ESM Table 2. Hazard ratios for incident diabetes comparing GAD65 antibody positive with GAD65 antibody negative groups, only including individuals with complete data on all covariates used in Model 3; the EPIC-InterAct study.

	**Model 1**					**Model 2**					**Model 3**				
	**N**	**HR**	**Lower**	**Upper**	**I^2 (%)**	**N**	**HR**	**Lower**	**Upper**	**I^2 (%)**	**N**	**HR**	**Lower**	**Upper**	**I^2 (%)**
GAD65 antibody$^+$ vs GAD65 antibody$^-$	12,835	2.05	1.52	2.77	38	12,835	2.00	1.45	2.76	42	12,835	2.19	1.56	3.07	43
"High" GAD65 antibody$^+$ vs GAD65 antibody$^-$	12,835	2.66	1.65	4.27	57	12,835	2.55	1.54	4.23	59	12,835	2.73	1.58	4.70	62
"Low" GAD65 antibody$^+$ vs GAD65 antibody$^-$		1.54	1.05	2.27	15		1.49	1.04	2.13	2		1.74	1.21	2.50	3

Model 1: age (as underlying time scale), sex, center; Model 2: age (as underlying time scale), sex, center, physical activity, smoking status, education; Model 3: age (as underlying time scale), sex, center, physical activity, smoking status, education, family history of diabetes. I^2 represents % of variability due to heterogeneity between countries. “High” GAD65 antibody$^+$ are defined as GAD65 antibody ≥167.5 U/ml and “Low” GAD65 antibody$^+$ are defined as GAD65 antibody ≥167.5 U/ml.

ESM Table 3. Associations of type 2 diabetes SNPs with GAD65 antibody positivity in the EPIC-InterAct sub-cohort

	SUBCOHORT	
	N	OR
rs10401969	12732	1.25
rs10758593	12779	1.14
rs10830963	12779	0.94
rs10923931	12779	1.02
rs11063069	12779	0.90
rs11257655	12779	1.02
rs12779790	12494	1.07
rs12899811	12779	1.11
rs12970134	12779	0.82
rs1535500	12494	0.95
rs163184	12779	0.99
rs16927668	12779	0.96
rs17168486	12779	1.06
rs17301514	12779	1.36
rs17762454	12494	0.98
rs2261181	12779	1.42
rs2334499	12779	1.07
rs243088	12779	0.91
rs2943640	12779	0.86
rs4299828	12779	0.91

rs4402960	12779	0.94
rs4458523	12577	0.91
rs459193	12779	1.01
rs4812829	12779	1.04
rs4865796	12494	0.93
rs516946	12779	0.98
rs6808574	12494	1.09
rs6975024	12494	0.83
rs7177055	12779	0.74
rs7756992	12779	1.27
rs780094	12779	0.87
**rs7903146**	**12779**	**1.10**
rs8090011	12494	1.00
rs9936385	12779	0.93
rs10203174	12694	0.98
rs10842994	12779	1.17
rs10886471	12261	1.09
rs11065756	12448	0.97
rs1111875	12779	1.05
rs11717195	12779	1.03
rs12427353	12779	0.86
rs12497268	12649	0.82
rs12571751	12779	1.07
rs13233731	12779	1.08
rs13389219	12779	1.06
rs1359790	12779	0.95
rs1496653	12779	1.00
rs1552224	12779	0.96
rs17106184	12425	1.05
rs17791513	12779	1.34

rs17867832	12707	1.06
rs1801282	12708	1.00
rs2028299	12494	0.87
rs2075423	12779	1.01
rs3802177	12779	0.98
rs391300	12494	0.99
rs4502156	12779	0.94
rs6795735	12779	1.14
rs6813195	12350	1.16
rs6819243	12761	1.53
rs6878122	12779	1.10
rs7403531	12494	1.12
rs7569522	12779	1.01
rs7845219	12779	1.00
rs7955901	12779	1.06
rs8182584	12779	1.14
rs849135	12779	0.95
rs7202877	12707	0.87

Odds ratios are per 1 unit (i.e. risk allele) of each SNP/variant.
Associations are estimated from logistic regression, adjusted for age and sex.
Models fit within each country; estimates combined across countries using random effects meta-analysis.
The rs7903146 (SNP at TCF7L2 locus) is highlighted in bold font.

ESM Table 4. Baseline characteristics of the subcohort, by GAD65 antibody status and tertile of type 1 diabetes-GRS; the EPIC-InterAct study*.

	Low/middle tertile		High tertile	
	GAD65 antibody^{-} n=8,295	GAD65 antibody^{+} n=149	GAD65 antibody^{-} n=4,231	GAD65 antibody^{+} n=104
Women (%)	61.5	63.8	62.7	66.4
Age (years)	52.2 (9.2)	52.2 (9.2)	52.5 (9.2)	54.0 (8.9)
BMI (kg/m^2)	26.0 (4.2)	25.5 (4.2)	25.9 (4.1)	25.7 (3.9)
Waist-hip ratio	0.85 (0.09)	0.85 (0.10)	0.85 (0.09)	0.85 (0.09)
Physical activity				

Inactive	23.1	29.3	22.4	16.5
Moderate inactive	33.3	36.1	33.6	37.9
Moderate active	22.7	19.1	23.8	23.3
Active	21.0	15.6	20.2	22.3
Highest school level				
None	6.9	10.2	7.6	6.7
Primary	32.4	31.3	32.7	38.5
Technical	24.6	24.5	24.3	20.2
Secondary	15.0	14.3	15.0	13.5

Further education	21.1	19.7	20.5	21.2
Smoking status				
Never	45.5	48.6	48.3	48.1
Former	27.7	26.7	25.8	25.0
Current	26.8	24.7	25.9	26.9
Family history of diabetes (yes)	18.3	6.8	19.8	19.6
GAD65 antibody (U/ml)	0.0 (0,0)	123.9 (80.4;257.9)	0.0 (0,0)	156.6 (99.0;747.8)
Type 1 diabetes risk score	0.5 (0.1)	0.5 (0.1)	0.7 (0.04)	0.7 (0.04)

Type 2 diabetes risk score	70.5 (5.7)	70.5 (5.6)	70.3 (5.7)	70.2 (4.9)

Values presented are percentages for categorical variables, mean (SD) for continuous variables except for GAD65 antibody, which has a skewed distribution, so median (IQR) is presented.

* Of the 15,802 members of the subcohort presented in Table 1 who all had GAD65 antibody status, 3,023 individuals did not have data for the type 1 diabetes genetic risk score, so are excluded from this table, which therefore includes N=12,779 individuals.

ESM Fig. 1. Receiver Operating Characteristic curve for GAD65 antibody levels, using the competition assay as the gold standard. Inserted panel shows GAD65 antibody levels plotted against percent inhibition.

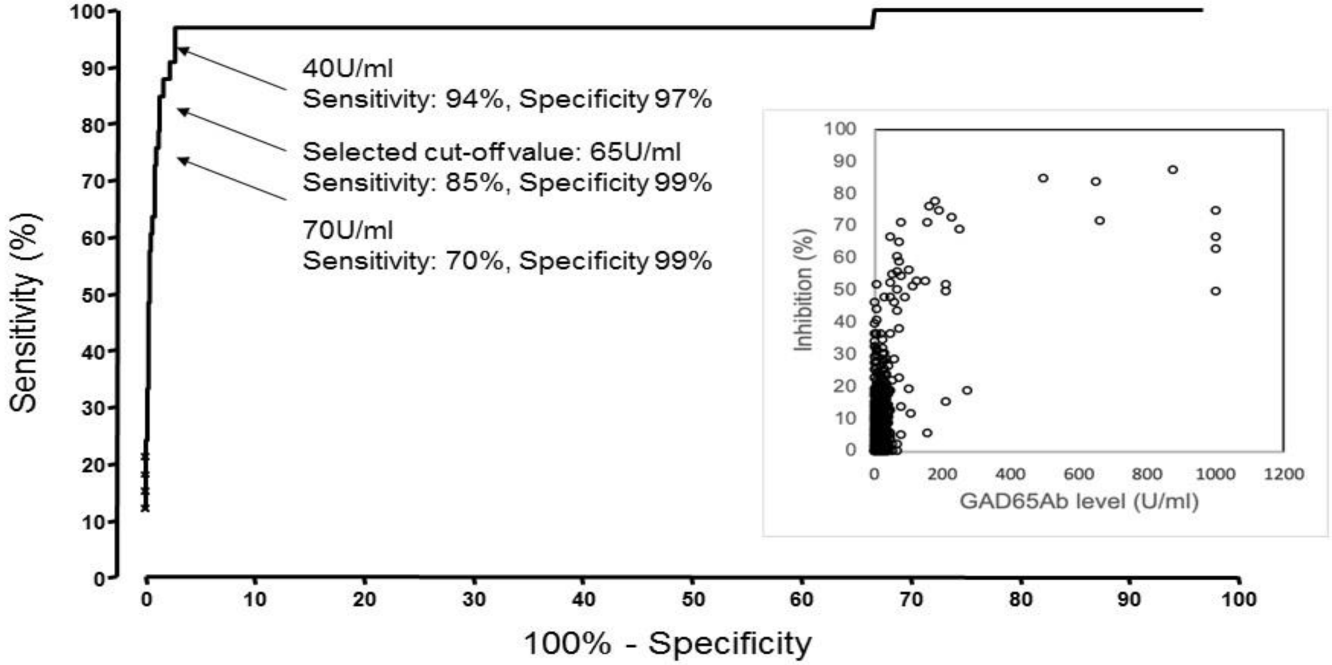

Supplement: Supplementary file 1 — (PDF 230 kb) [file 125_2019_5016_MOESM1_ESM.pdf]
